# Reactive hyperemia index (RHI) and cognitive performance indexes are associated with histologic markers of liver disease in subjects with non-alcoholic fatty liver disease (NAFLD): a case control study

**DOI:** 10.1186/s12933-018-0670-7

**Published:** 2018-02-16

**Authors:** Antonino Tuttolomondo, Salvatore Petta, Alessandra Casuccio, Carlo Maida, Vittoriano Della Corte, Mario Daidone, Domenico Di Raimondo, Rosaria Pecoraro, Roberto Fonte, Anna Cirrincione, Rita Zafonte, Daniela Cabibi, Calogero Cammà, Vito Di Marco, Anna Licata, Franco Magliozzo, Giulio Marchesini, Giovanni Merlino, Antonio Craxì, Antonio Pinto

**Affiliations:** 1 U.O.C di Medicina Interna con Stroke Care, Dipartimento Biomedico di Medicina Interna e Specialistica (Di.Bi.M.I.S), University of Palermo, P.zza delle Cliniche n.2, Palermo, 90127 Italy; 2 Sezione di Gastroenterologia e Epatologia, Dipartimento Biomedico di Medicina Interna e Specialistica (Di.Bi.M.I.S), University of Palermo, Palermo, Italy; 3 Dipartimento di Scienze per la Promozione della Salute e Materno Infantile, University of Palermo, Palermo, Italy; 4 Medicina Generale Palermo, Palermo, Italy; 5 Cattedra di Anatomia Patologica, University of Palermo, Palermo, Italy; 6 Dipartimento di Scienze Mediche e Chirurgiche, “Alma Mater Studiorum”, University of Bologna, Bologna, Italy

**Keywords:** Non-alcoholic fatty liver disease, Histopathology, Liver fibrosis

## Abstract

**Background:**

No study evaluated vascular health markers in subjects with non-alcoholic fatty liver disease (NAFLD) through a combined analysis of reactive hyperemia peripheral arterial tonometry (RH-PAT) and arterial stiffness indexes.

**Aim of the study:**

We aimed to assess whether NAFLD and its histological severity are associated with impairment of arterial stiffness and RH-PAT indexes in a mixed cohort of patients with biopsy-proven NAFLD.

**Materials and methods:**

The Kleiner classification was used to grade NAFLD grade. Pulse wave velocity (PWV) and augmentation index (Aix) were used as markers of arterial stiffness, whereas endothelial function was assessed using reactive hyperemia index (RHI). The mini-mental state examination (MMSE) was administered to test cognitive performance.

**Results:**

80 consecutive patients with biopsy-proven NAFLD and 83 controls without fatty liver disease. NAFLD subjects showed significantly lower mean RHI, higher mean arterial stiffness indexes and lower mean MMSE score. Multivariable analysis after correction for BMI, dyslipidaemia, hypertension, sex, diabetes, age and cardiovascular disease showed that BMI, diastolic blood pressure and RHI are significantly associated to NAFLD. Simple linear regression analysis showed among non-alcoholic steatohepatitis (NASH) subjects a significant negative relationship between ballooning grade and MMSE and a significant positive association between Kleiner steatosis grade and augmentation index.

**Conclusions:**

Future research will be addressed to evaluate the relationship between inflammatory markers and arterial stiffness and endothelial function indexes in NAFLD subjects. These study will evaluate association between cardiovascular event incidence and arterial stiffness, endothelial and cognitive markers, and they will address the beneficial effects of cardiovascular drugs such as statins and ACE inhibitors on these surrogate markers in NAFLD subjects.

## Introduction

Liver is a target organ in metabolic syndrome (MS) patients in which it manifests itself with non-alcoholic fatty liver disease (NAFLD) spanning steatosis via steatohepatitis and cirrhosis (1.2). Non-alcoholic fatty liver disease and non-alcoholic steatohepatitis (NASH) have been reported as associated with cardiovascular events [1–3].

Several useful surrogates in the clinical setting include Framingham Risk Score, carotid artery intima-media thickness, high sensitivity C-reactive protein (hsCRP), visceral and mediastinal fat, and coronary calcium scores [4, 5].

Forearm flow-mediated vasodilation (FMD) is a commonly used method for the assessment of endothelial dysfunction [6]. However, findings offered by FMD can be very different owing to technical issues thus this method has been reported as not standardized [7].

Reactive hyperemia peripheral arterial tonometry (RH-PAT) is a new method useful to measure endothelial dysfunction that has been reported by Kuvin et al. [8]. This method is noninvasive, and quantitative and it offers a digital measurement of hyperemic response. Framingham Heart Study reported that reactive hyperemia index (RHI) is inversely correlated with various cardiovascular risk factors [9].

Adjunctive surrogates for cardiovascular risk more recently validated by some studies [10, 11] are arterial stiffness and endothelial function indexes.

Augmentation pressure and pulse wave velocity (PWV) have been reported as surrogate markers for cardiovascular disease [10]. They are both two markers of endothelial dysfunction and vascular damage [11] and some studies analyzed arterial stiffness indexes in NAFLD patients [12–14].

Vlachopoulos et al. [12] reported that NAFLD subjects showed significantly higher carotid-femoral pulse wave velocity and reduced flow-mediated dilatation (FMD) and that at multivariable analysis, presence of NAFLD was an independent determinant of both PWV and FMD. In another study Salvi et al. [13] reported that patients with NAFLD and MS PWV values were significantly higher in comparison to controls and to NAFLD subjects without MS.

Nonetheless, only a study [15] analyzed endothelial dysfunction in these subjects using flow mediated dilation and flow-mediated vasoactivity (FMV) findings. Authors reported that NAFLD was associated with a percent FMV in the lower tertile after adjustment for age, sex, body mass index, and insulin resistance and that among NAFLD patients, low FMV was associated with nonalcoholic steatohepatitis.

Vascular disease seems to be important in cognitive impairment and cerebrovascular disease, including white matter lesions and silent infarcts that may lead to a specific pattern of cognitive impairment. It explains that cognitive function indexes such as mini-mental state examination (MMSE) scores could be putative to be considered a surrogate vascular marker.

Thus, the evaluation by means the analysis of RH-PAT indexes, arterial stiffness markers and cognitive performance could offer a more complete and useful information with regard of the vascular health status of NAFLD patients.

No study, to the best of our knowledge, has analyzed endothelial dysfunction in subjects with NAFLD through an analysis of RH-PAT indexes. Furthermore no study analyzed the relationship between histological severity of NAFLD and arterial stiffness and RH-PAT indexes and no study evaluated cognitive indexes as surrogate vascular markers in association with other surrogate markers such as arterial stiffness and endothelial function indexes, whereas only one study evaluated the relationship between cognitive function and NAFLD [14].

Considering the link between NAFLD, its related metabolic changes and cardiovascular risk, to assess whether NAFLD and its histological severity is related to the impairment of some surrogate markers of vascular impairment, we designed a study with a principal aim:

1. To evaluate arterial stiffness and RH-PAT indexes in a mixed cohort of patients with biopsy-proven NAFLD in comparison with subjects without fatty liver and with regard to NASH presence; and two secondary aims:
2. To evaluate cognitive performance measured by MMSE in a mixed cohort of patients with biopsy-proven NAFLD in comparison with subjects without fatty liver and with regard to NASH presence;
3. To analyze the relationship between hepatic histological findings of NAFLD and surrogate markers of vascular impairment (arterial stiffness and RH-PAT indexes and cognitive performance index).

## Patients and methods

### Patients

We recruited all NAFLD patients admitted to the Gastrointestinal & Liver Unit of Palermo University Hospital from June 2014 to December 2015.

Inclusion criteria were: (1) a histological diagnosis of NAFLD on a liver biopsy obtained less than 6 months before enrollment.

A pre-biopsy evaluation of NAFLD was based on these findings: increased serum levels of alanine transaminase (ALT) for at least 6 months and alcohol consumption of < 20 g/day in the previous year (also confirmed by a questionnaire). Liver ultrasound was performed in all groups (study group and control) and patients were also recruited by ultrasound (US) finding of steatosis, and a liver stiffness value > 6 kPa.

Exclusion criteria were: (1) steatosis, (2) decompensated cirrhosis; (3) hepatocellular carcinoma; (4) liver disease of different or mixed etiology (alcoholic, viral, autoimmune Wilson’s disease, hemochromatosis, α1-antitrypsin deficiency); (5) human immunodeficiency virus infection; [6] regular use of steatosis-inducing drugs (steroid, amiodarone, tamoxifen, etc.).

Subjects without NAFLD were referred from general practitioners or they were recruited among patients admitted to Internal Medicine and Stroke Care ward of Policlinico Universitario “P. Giaccone”, University of Palermo, Italy. All the subjects enrolled as controls had no history of previous cardiovascular or cerebrovascular disease or metabolic disease or acute disease such as acute myocardial infarction (AMI) or stroke or infections or deep venous thrombosis. They were metabolic and cardiovascular disease free subjects referred from general practioners or admitted to our department for another disease else those indicated as exclusion criteria in the same period.

Among 209 that were evaluated, 120 had no steatosis but satisfied all the criteria indicated above. They had no previous history of previous cardiovascular events, no history of liver disease, no evidence of viral infection (anti-HCV, anti-HIV, and HBsAg negativity), alcohol consumption > 20 g/day during the previous year (evaluated by a specific questionnaire), normal alanine transaminase values (< 37 UI/l), no ultrasound (US) finding of steatosis, and a liver stiffness value < 6 kPa.

### Consent for publication

All enrolled patients released a consent to publish from the participant (or legal parent or guardian for children) to report individual patient data.

### Availability of data and material

All data and material are available on figshare.

#### Clinical and laboratory assessment

Clinical and anthropometric data were collected at the time of enrollment. Subjects were classified as normal weight (BMI 18.5–24.9 kg/m^2^), overweight (BMI 25–29.9), obese (BMI ≥ 30). The diagnosis of arterial hypertension was based on the following criteria: systolic blood pressure ≥ 140 mmHg and/or diastolic blood pressure ≥ 90 mmHg (measured three times within 30 min, in the sitting position and using a brachial sphygmomanometer), or use of blood-pressure-lowering agents [16]. The diagnosis of type 2 diabetes was based on the revised criteria of the American Diabetes Association, using a value of fasting blood glucose ≥ 126 mg/dl [17]. In patients with a previous diagnosis of type 2 diabetes, current therapy with insulin or oral hypoglycemic agents was documented. A 12-h overnight fasting blood sample was drawn at the time of enrollment to determine the serum levels of ALT, total cholesterol, HDL-cholesterol, triglycerides, plasma glucose, insulin (for NAFLD only), and platelet count (for NAFLD). Dyslipidemia was defined as Triglyceride (TG) level ≥ 150 mg/dl and total cholesterol levels > 200 mg/dl. We also performed HDL-levels categorization according to a gender-specific threshold for HDL (40 mg/dl for male and 50 mg/dl for females) [18].

#### Ultrasound assessment

Ultrasound assessment was performed on fasting subjects, on the day of liver biopsy for NAFLD, and on the day of enrollment of the controls, by one operator trained in ultrasound techniques and dedicated to liver examination. A real-time Hitachi H21 apparatus with a 2–5 MHz, convex, multi-frequency probe was used. Presence of hepatic steatosis was defined by detection of Bright Liver Echo pattern (BLEP), i.e. fine, packed and high amplitude echoes, with consequent brightness of liver, increase in liver–kidney contrast and possible evidence of vascular blurring and deep attenuation signs [19].

#### Liver stiffness measurement (LSM)

Transient elastography was performed with the FibroScan (Echosens, Paris, France) medical device, using the M probe (also called standard probe), following the ultrasound examination, by trained operators who had previously performed at least 300 determinations in patients with chronic liver disease. As recently reported in the literature [20], we classified LSM examinations into three reliability categories: “very reliable” (IQR/M ≤ 0.10), “reliable” (0.10 < IQR/M ≤ 0.30, or IQR/M > 0.30 with LSM median < 7.1 kPa), and “poorly reliable” (IQR/M > 0.30 with LSM median ≥ 7.1 kPa). “Poorly reliable” results were excluded from the analysis.

### Assessment of histology

Slides of NAFLD were coded and read by one pathologist (D.C.), who was unaware of the patients’ identity and history. A minimum length of 15 mm of biopsy specimen or the presence of at least 10 complete portal tracts was required [21]. Steatosis was assessed as the percentage of hepatocytes containing fat droplets (minimum 5%), and evaluated as a continuous variable. The Kleiner classification [22] was used to grade steatosis, lobular inflammation, and hepatocellular ballooning, and to stage fibrosis from 0 to 4 [23]. NASH was considered to be present when steatosis, ballooning, and lobular inflammation were present.

### Pulse wave velocity (PWV) measurement

Carotid-femoral PWV was measured in the supine position using an automatic device (SphygmoCor version 7.1) that measured the time delay between the rapid upstroke of the carotid and femoral artery pulse waves. The distance between the 2 arterial points was measured on the surface of the body using a tape measure. PWV was calculated as the distance traveled by the arterial pulse wave (meters) divided by the time delay between the 2 arterial points (seconds), thus expressed as meters per second.

### Pulse wave analysis

Applanation tonometry was used to record radial artery pressure waveform continuously, and mean values of ≥ 2 screens of pulse waves of good quality were used for analysis. From the collected data, an averaged radial pressure waveform was generated and a corresponding aortic pressure waveform and BP was calculated by the validated transfer function (SphygmoCor version 7.1). The aortic pressure waveform was used to calculate the AIx (difference in height between the first and second systolic peaks expressed as a percentage of PP).

### RH-PAT

The principle of RH-PAT has been described previously by some researchers [15]. Briefly, blood pressure cuff was placed on 1 upper arm, while the contralateral arm served as a control. PAT probes were placed on 1 finger of each hand. After a 5 min equilibration period, the cuff was inflated to 60 mmHg above the systolic pressure or 200 mmHg for 5 min and then deflated to induce reactive hyperemia.

The RH-PAT data were digitally analyzed online (Endo-PAT2000 software version 3.0.4). The RH-PAT index reflects the extent of reactive hyperemia and was calculated as the ratio of the average amplitude of PAT signal over 1 min starting 1.5 min after cuff deflation (control arm, A; occludedarm, C) divided by the average amplitude of PAT signal of a 2.5 min time period before cuff inflation (baseline) (control arm, B; occluded arm, D). Thus RH-PAT index (RHI) = (C/D)/(A/B) × baseline correction.

### Assessment of cognitive function (mini-mental state examination)

The mini-mental state examination (MMSE) a tool that can be used to systematically and thoroughly assess cognitive function was administered. It is an 11-question measure that tests 5 areas of cognitive function (orientation, registration, attention and calculation, recall, and language). The maximum score is 30 whereas an MMSE value < 24 (23 or lower) is indicative of cognitive impairment [24].

### Statistical analysis

Statistical analysis of quantitative and qualitative data, including descriptive statistics, was performed for all items. Continuous data are expressed as mean ± SD, unless otherwise specified. Baseline differences between groups were assessed by the Chi square test or Fisher exact test, as needed for categorical variables, and by the independent Student t test for continuous parameters.

The analysis of variance (ANOVA) was performed for parametric variables in univariable and multivariable model to evaluate the difference between NASH vs no NASH patients. In both models the data were adjusted for BMI, dyslipidaemia, hypertension, sex, diabetes, cardiovascular disease and age.

Simple linear regression analysis was used to assess histological hepatic damage markers (Kleiner classification indexes) independently associated with vascular damage indexes in NASH and no NASH patients, respectively. B coefficients (B) and their 95% confidence intervals (CIs) were also calculated and adjusted for BMI, dyslipidaemia, hypertension, sex, diabetes, cardiovascular disease and age. In the model, the dependent variables were RHI, PWV, Aix and MMSE. Data were analyzed by the SPSS Software 22.0 version (IBM Corp., Armonk, NY, USA). All p-values were two-sided, and p-values less than 0.05 were considered statistically significant.

## Results

The study involved 80 consecutive patients with biopsy-proven NAFLD and 83 controls without a fatty liver disease. General and laboratory variables of patients with NAFLD and controls are listed in Table 1.

**Table 1:** Demographic, laboratory and clinical variables in NAFLD subjects and controls

Variable	NAFLD subjects (n = 80)	Controls (n = 83)	p
Age (years) (mean ± SD)	53.7 ± 12.9	51.7 ± 17.2	0.401
Weight (kg) (mean ± SD)	78.9 ± 17.7	71.3 ± 13.4	*0.002*
BMI (kg/m^2^) (mean ± SD)	28.7 ± 5.6	25.3 ± 3.8	*0.005*
SBP (mm/Hg) (mean ± SD)	121.7 ± 13.7	122.7 ± 16.3	0.655
DBP (mm/Hg) (mean ± SD)	72.7 ± 9.5	75.4 ± 12.7	0.127
Hypertension (n/%)	28 (35.0)	13 (15.7)	*0.006*
Diabetes (n/%)	31 (38.8)	7 (8.4)	*0.005*
Dyslipidaemia (n/%)	15 (18.8)	14 (16.9)	0.839
Previous cardiovascular diseases	3 (3.75)	0	0.341
Previous cerebrovascular events	0	0	0.417
RHI (mean ± SD)	1.85 ± 0.5	2.20 ± 0.6	*0.005*
Aix (%) (mean ± SD)	144.3 ± 27.2	131.4 ± 31.4	*0.006*
PWV (mt/s) (mean ± SD)	10.46 ± 2.1	9.61 ± 2.30	*0.015*
MMSE (mean ± SD)	26.9 ± 1.6	28.0 ± 1.4	*0.005*
Liver stiffness, kPa	9.37 ± 7.7	4.5 ± 0.4	*0.005*
NASH patients (n/%)	52		
Histology (%)	52 (65)		
Kleiner steatosis grade 0–3 (mean/SD)			
1 (n/%)	36 (45)		
2 (n/%)	22 (27.5)		
3 (n/%)	22 (27.5)		
Lobular inflammation grade 0–3 (mean/SD)			
0 (n/%)	2 (2.5)		
1 (n/%)	45 (56.3)		
2 (n/%)	31 (38.8)		
3 (n/%)	2 (2.5)		
Hepatocellular ballooning 0–2 (mean/SD)			
0 (n/%)	23 (28.7)		
1 (n/%)	35 (43.8)		
2 (n/%)	22 (27.5)		
Stage of fibrosis (0–4) (mean/SD)			
0 (n/%)	18 (22.5)		
1 (n/%)	25 (31.3)		
2 (n/%)	14 (17.5)		
3 (n/%)	17 (21.3)		
4 (n/%)	6 (7.5)		

Liver stiffness: measured by transient elastography (TE) (fibroscan)

Significant p values are indicated in Italics

*SBP* systolic blood pressure, *DBP* diastolic blood pressure, *BMI* body mass index, *Aix*: augmentation index, *PWV* pulse wave velocity, *RHI* reactive hyperemia index, *MMSE* mini-mental state examination, *kPa* kilopascal

NAFLD subjects in comparison to controls had a higher BMI (28.7 ± 5.6 vs. 25.3 ± 3.8; p = 0.005) and liver stiffness (9.37 ± 7.7 vs 4.5 ± 0.4 kPa) and they were more likely to have hypertension (35% vs. 15.7%; p = 0.006) and diabetes (38.8% vs. 8.4%, p = 0.005).

NAFLD subjects in comparison to controls also had a significantly lower mean RHI (1.85 ± 0.5 vs. 2.20 ± 0.6; p = 0.005) and higher mean arterial stiffness indexes such as Aix (144.3 ± 27.2 vs. 131.4 ± 31.4, p = 0.006) and PWV (10.46 ± 2.1 m/s vs. 9.61 ± 2.30 m/s; p = 0.015). MMSE was available for 139 patients (74 with and 65 without NAFLD), with similar characteristics compared to the entire cohort (data not shown).

NAFLD patients had a lower mean MMSE score (26.9 ± 1.6 vs. 28.0 ± 1.36; p = 0.005) in comparison with patients without NAFLD.

At univariable analysis we observed a significant relationship between BMI (p = 0.004), diastolic blood pressure (p = 0.019), Aix (p = 0.018), MMSE (p < 0.0001), RHI (p = 0.007) and NAFLD, whereas at multivariable analysis after correction for BMI, dyslipidaemia, hypertension, sex, diabetes and age and cardiovascular disease only BMI (p = 0036), diastolic blood pressure (p = 0.048), MMSE (p < 0.0001) and RHI (p = 0.032) remained significantly associated to NAFLD (see Table 2).

**Table 2:** Univariate and multivariate analysis of relationship between clinical variables and NAFLD

Dependent variables	F	Sign.
Univariable analysis		
Age	0.011	0.915
BMI	8.452	*0.004*
SBP	3.854	0.051
DBP	5.581	*0.019*
Aix	5.751	*0.018*
PWV	2.223	0.138
MMSE	20.069	*< 0.0001*
RHI	7.423	*0.007*
Multivariable analysis		
BMI	4.512	*0.036*
SBP	3.688	0.057
DBP	3.977	*0.048*
Aix	1.615	0.206
MMSE	24.999	*<0.0001*
RHI	4.722	*0.032*

Adjusted for BMI, dysilipidaemia, hypertension, sex, diabetes and age

Significant p values are indicated in Italics

In NAFLD subjects we observed no significant difference with regard to vascular damage markers and MMSE score accordingly to NASH presence or absence (see Table 3).

**Table 3:** Clinical variables of NAFLD patients according to NASH presence

Variable	No NASH subjects (n = 28)	NASH subjects (n = 52)	p
Age (years) (mean ± SD)	51.5 ± 12.8	54.9 ± 12.8	0.258
Weight (kg) (mean ± SD)	78.6 ± 19.9	79.3 ± 15.3	0.850
BMI (kg/m^2^) (mean ± SD)	27.6 ± 6.3	29.3 ± 5.1	0.812
SBP (mm/Hg) (mean ± SD)	120.1 ± 13.1	122.5 ± 14.1	0.443
DBP (mm/Hg) (mean ± SD)	72.3 ± 9.6	72.9 ± 9.6	0.771
Gender M/F	15/13	25/27	0.815
Hypertension (n/%)	8 (28.6)	20 (38.5)	0.464
Diabetes (n/%)	9 (32.1)	22 (42.3)	0.472
Dyslipidaemia (n/%)	4 (14.3)	11 (21.1)	0.557
Previous cardiovascular diseases	0(0)	3 (5.8)	0.309
Previous cerebrovascular events	0	0	/
RHI (mean ± SD)	1.8 ± 0.51	1.9 ± 0.52	0.670
Aix (%) (mean ± SD)	141.1 ± 25.4 26.8 ± 12.3	146.1 ± 28.1 29.6 ± 12.2	0.338 0.429
PWV (mt/s) (mean ± SD)	10.4 ± 1.9	10.4 ± 2.2	0.971
MMSE (mean ± SD)	27.5 ± 2.0	27.3 ± 2.9	0.765
Liver stiffness, kPa	6.7 ± 2.7	11.5 ± 8.0	*0.003*
Histology (%)			
Kleiner steatosis grade 0–3			
1 (n/%)	16 (57.1)	20 (38.5)	
2 (n/%)	7 (25.0)	15 (28.8)	0.271
3 (n/%)	5 (17.9)	17 (32.7)	
Lobular inflammation grade 0–3			
0 (n/%)	2 (7.1)	0	
1 (n/%)	23 (82.1)	22 (42.3)	
2 (n/%)	2 (7.1)	29 (55.8)	*< 0.0001*
3 (n/%)	1 (3.6)	1 (1.9)	
Hepatocellular ballooning (0–2)			
0 (n/%)	11 (39.3)	12 (23.1)	
1 (n/%)	15 (53.6)	20 (38.4)	*0.010*
2 (n/%)	2 (7.1)	20 (38.4)	
Stage of fibrosis (0–4)			
0 (n/%)	11 (39.3)	7 (13.5)	
1 (n/%)	14 (50.0)	11 (21.1)	
2 (n/%)	3 (10.7)	11 (21.1)	*< 0.0001*
3 (n/%)	0	17 (32.7)	
4 (n/%)	0	6 (11.5)	

Significant p values are indicated in Italics

*SBP* systolic blood pressure, *DBP* diastolic blood pressure, *BMI* body mass index, *Aix*: augmentation index, *PWV* pulse wave velocity, *RHI* reactive hyperemia index, *MMSE* mini-mental state examination, *kPa* kiloPascal

In NAFLD subjects we also observed no significant difference with regard of vascular damage markers an MMSE score accordingly to fibrosis severity (see Table 4).

**Table 4:** Clinical variables of NAFLD patients according to NASH and severity of fibrosis

Variable	Subjects with fibrosis 0–1 (n = 43)	Subjects with fibrosis ≥ 2 (n = 37)	p
Age (years) (mean ± SD)	50.2 ± 11.9	57.8 ± 12.9	0.008
Weight (kg) (mean ± SD)	75.3 ± 14.0	83.2 ± 20.4	0.045
BMI (kg/m^2^) (mean ± SD)	27.4 ± 5.1	30.3 ± 5.7	0.017
SBP (mm/Hg) (mean ± SD)	117.8 ± 11.7	126.2 ± 14.6	0.006
DBP (mm/Hg) (mean ± SD)	71.9 ± 8.4	73.6 ± 10.8	0.440
Gender M/F	24/19	16/21	0.262
Hypertension (n/%)	11 (25.6)	17 (45.9)	0.057
Diabetes (n/%)	10 (23.3)	21 (56.8)	0.002
dyslipidemia (n/ %)	7 (16.3)	8 (21.6)	0.542
Previous cardiovascular diseases	0 (0)	3 (8.1)	0.057
Previous cerebrovascular events	0	0	/
RHI (mean ± SD)	1.9 ± 0.54	1.8 ± 0.47	0.211
Aix (%)	139.8 ± 29.4	149.7 ± 23.6	0.103
(mean ± SD)	25.8 ± 13.2	31.9 ± 10.4	0.027
PWV (mt/s) (mean ± SD)	10.0 ± 1.9	10.9 ± 2.3	0.043
MMSE (mean ± SD)	27.8 ± 2.2	26.9 ± 2.9	0.131
Liver stiffness, kPa	7.5 ± 3.6	12.6 ± 8.9	0.001
Histology (%)			
Kleiner steatosis grade 0–3			
1 (n/%)	29 (67.4)	7 (18.9)	
2 (n/%)	7 (16.3)	15 (40.5)	< 0.0005
3 (n/%)	7 (16.3)	15 (40.5)	
Lobular inflammation grade 0–3			
0 (n/%)	2 (4.6)	0	
1 (n/%)	36 (83.7)	9 (24.3)	
2 (n/%)	4 (9.3)	27 (73.0)	*< 0.0005*
3 (n/%)	1 (2.3)	1 (2.7)	
Hepatocellular ballooning (0–2)			
0 (n/%)	21 (48.8)	2 (5.4)	
1 (n/%)	20 (46.5)	15 (40.5)	*< 0.0005*
2 (n/%)	2 (4.6)	20 (54.0)	

Significant p values are indicated in Italics

*SBP* systolic blood pressure, *DBP* diastolic blood pressure, *BMI* body mass index, *Aix* augmentation index, *PWV* pulse wave velocity, *RHI* reactive hyperemia index, *MMSE* mini-mental state examination, *kPa* kiloPascal

At simple linear regression, after correction for BMI, dyslipidaemia, hypertension, sex, diabetes, age and cardiovascular disease, analysis of the relationship between hepatic histological findings and vascular damage markers, showed a significant negative relationship linking MMSE and Kleiner ballooning grade [B = − 1.344 (95% CI = − 2.650 to 0.037); p = 0.044] and a significant positive relationship linking Aix and Kleiner steatosis grade [B = 4.957 (95% CI = 1.385–8.529); p = 0.008] in NAFLD subjects with NASH (see Table 5).

**Table 5:** Simple linear regression analysis of variables associated with vascular damage indexes (RHI, AIX, PWV and MMSE) in NAFLD subjects with and without NASH

Vascular damage indexes	Variable	NASH		No-NASH
		B (95% CI)	p	
PWV	–	–		–
AIX	Kleiner steatosis grade	4.957 (1.385 to 8.529)	0.008	–
RHI	–	–		–
MMSE	Kleiner balloning grade	− 1.344 (− 2.650 to − 0.037)	0.044	–

Adjusted for BMI, dysilipidemia, hypertension, sex, diabetes and age and cardiovascular diseases

No significant relationship was observed between hepatic histological findings and vascular damage markers among NAFLD subjects without NASH.

## Discussion

In a mixed cohort of patients with NAFLD and controls without fatty liver, we report that subjects with NAFLD had significantly lower RHI values and higher mean arterial stiffness indexes such as Aix and PWV, and that NAFLD subjects also had lower mean MMSE scores in comparison with control subjects without NAFLD.

To the best of our knowledge, no study has yet addressed the relationship between endothelial dysfunction markers evaluated by reactive hyperemia peripheral arterial tonometry (RH-PAT) and NAFLD. Some studies analyzed arterial stiffness indexes in subjects with NAFLD [25–27] and just one study evaluated cognitive performance in NAFLD patients [28]. Thus, our findings of impaired vascular damage markers in subjects with NAFLD could appear as novel.

The study by Leite et al. [25] showed that diabetic patients with NAFLD are more likely to have high or increasing aortic stiffness that predicted the development of advanced liver fibrosis transient elastography. The study by Sunbul et al. [27] analyzed patients with biopsy-proven NAFLD whereas the study by Seo et al. [28] analyzed subjects with ultrasound defined NAFLD.

In particular the study of Sunbul [27] showed that NAFLD patients compared with controls, had significantly higher PWV and AIx values. Nevertheless, in this previous study authors analyzed the relationship between arterial stiffness indexes and only liver fibrosis scores. Thus, our current findings obtained after an analysis of the relationship between arterial stiffness indexes and the full range of hepatic histological changes in NAFLD subjects may be considered a potential original result.

Endothelial dysfunction is one of the earliest events in the pathogenetic process leading to the atherosclerotic disorders [29], and it contributes to the progression of disease through inflammation and thrombosis [30–32].

Studies using the EndoPAT have shown that the RHI score reflects NO-bioavailability [33]. The RHI correlates with the measurement of endothelial vasodilator function in the coronary arteries [34] and with brachial FMD [35]. Patients with a higher degree of cardiovascular involvement exhibit a lower score [36–38]. Notably, RHI values appear to be predictive of cardiovascular outcomes [39–41].

Thus our findings of lower values of RHI in subjects with NAFLD may be suggestive of the raised cardiovascular risk of patients with NAFLD due to a higher prevalence of endothelial dysfunction.

Multivariable analysis of association linking clinical variables and NAFLD showed the significant predictive role of RHI and MMSE. It is possible to speculate that endothelial dysfunction and impaired cognitive performance may represent a potential hallmark of NAFLD and that endothelial function and cognitive markers could be employed as putative indicators of early vascular involvement in this clinical context.

A recent study reported that NAFLD was independently associated with lower cognitive performance independent of CVD and its risk factors [14].

Our finding of lower MMSE values in subjects with NAFLD may be indicative of a lower degree of cognitive performance even in absence of MMSE scores indicating a clinical cognitive dysfunction, in subjects with higher degree of arterial stiffness and lower degree of endothelial mediated vasodilation. Consistent with our findings a recent study **[**51**]** showed that a MMSE cut score to 27 resulted in an optimal balance of sensitivity and specificity with an overall correct classification rate of 90% in a cognitively impaired group (dementia and MCI), thus indicating that a cut-score of 27 might be more appropriate.

Evidence from epidemiology and pathology studies indicates that damage to the vascular system is associated with an increased risk of many types of cognitive impairment [42–44]. Although we have not observed mean MMSE values indicating a cognitive dysfunction in both groups of subjects, our findings of lower MMSE mean scores observed in NAFLD patients (mean MMSE 26.9) and the negative relationship observed between ballooning grade and MMSE may represent a further issue confirming the role of metabolic disturbances linked to NAFLD also in clinical setting of cognitive performance. Nevertheless, we observed no significant association between cognitive performance and histological findings in NAFLD patients without NASH and only a significant negative association between ballooning grade and MMSE in NAFLD subjects with NASH. This finding is novel since that no previous study has not yet reported any relation between histological severity of NAFLD and cognitive performance. Our group recently reported [44] that white matter lesions (WMLs) are not associated with the presence of NAFLD but with its histological severity. This finding may also suggest an alternative pathogenic hypothesis to explain subclinical lower cognitive performance of NAFLD subjects such as small-artery remodeling and direct vascular cognitive impairment (VCI) well expressed by progressive arterial stiffening and endothelial dysfunction [45–50]. These vascular changes are probably related to metabolic disorders underlying NAFLD presence.

Previous studies from our group reported an higher degree of organ damage expressed as the diabetic foot [51], a vascular impairment [52, 53] and the Obstructive Sleep Apnea syndrome (OSAS) [54] in subjects with MS.

Overweight and obesity and associated co-morbidities are established cardiovascular (CV) risk. Endothelial dysfunction (ED) is the initial vascular damage, which precedes fatty streak known to initiate atherosclerosis a progressive disease which often culminates as a sudden cardiovascular event [55].

Arterial stiffness and MS have been reported as associated with cognitive impairment in elderly people. A recent study reported that age (older than 60 years) and MS play a synergistic role in cognitive decline pathogenesis in the elderly population [56].

Obstructive sleep apnea (OSA) and type 2 diabetes (T2D) are also associated with endothelial dysfunction [57], whereas increasing age, liver fat, and triglyceride are all related to increased aortic stiffness in adults [58] and a recent study indicates that NAFLD and MS have a synergistic impact on the subclinical atherosclerosis [59, 60].

Furthermore, a recent study reported that nonalcoholic fatty liver disease is associated with a smaller total cerebral brain volume, indicating a possible link between hepatic steatosis and brain aging [61].

These factors may represent possible explanation of our findings of a higher degree of vascular impairment in subjects with NAFLD.

Our findings consistent with previous study [62] underlined that the reported higher degree of arterial stiffness in NAFLD patients compared with controls might be related to the higher prevalence of cardiometabolic risk factors in NAFLD patients.

Thus, in NAFLD clinical setting liver and vessels represent parallel end-organ damage target of MS and insulin resistance.

This issue is furtherly confirmed by our findings about differences among NAFLD subjects in relation to NASH presence and fibrosis severity that showed no difference with regard of RHI and arterial stiffness markers in the two groups of subjects (NASH vs non NASH).

This is an interesting finding probably due to the fact the NAFLD subjects with NASH and those without NASH and subjects categorized in relation of fibrosis severity show similar prevalence of cardiovascular risk factors such as hypertension and diabetes and they also show not significant difference with regard of the mean value of arterial blood pressure and plasma cholesterol levels.

Nevertheless, although the presence of NASH does not seem significantly associated to a higher degree of vascular damage in patients with NAFLD our findings report a peculiar relationship between some histologic hepatic findings and vascular damage markers only in NASH subjects. Simple logistic regression analysis showed a negative association between ballooning grade and MMSE in NASH patients and a positive association has been observed in NASH subjects between steatosis grade and augmentation index.

Arterial stiffness is due to a complex interplay between dynamic or constant forces and structural and cellular components of the vascular wall such as hormones, salt and glucose aging and blood pressure [63, 64]. Resiliency and compliancy of the vascular wall depend on two proteins: collagen and elastin. The presence of such molecules in arterial wall is maintained by a constant interplay of production and degradation. An inflammatory milieu can trigger an impairment of this balance, driving to overproduction of altered collagen and impaired presence of elastin, leading to increased arterial stiffness [56]. Arterial hypertension and diabetes mellitus and ageing represent the main causes of these vascular alterations that worsen artery stiffening.

We observed no difference in main cardiovascular risk factor between NAFLD subjects with NASH and those without NASH, thus our findings of a significant relation between two histologic hepatic markers such as ballooning and steatosis grade could underline a possible prevalent role of a inflammatory milieu in arterial stiffening and cerebral microvascular disease pathogenesis. Many studies evaluated if NAFLD contributed to other outcomes, such as cardiovascular mortality; and most of them demonstrated an association, but no causality could be shown [65–67]. In this light liver disease and atherogenesis might be mediated by inflamed visceral adipose tissue: In this pathogenetic scenario, nonalcoholic steatohepatitis and mainly NASH presence might be related to the presence of vascular disease in two ways: first, through the production of some inflammatory, prothrombotic and oxidative-stress mediators and, second, by means the direct involvement of nonalcoholic fatty liver disease in insulin resistance mechanism and atherogenic dyslipidemia.

## Limitations

The main limitation of this study lies in its cross-sectional nature, unable to identify pathogenic mechanisms linking vascular damage and liver fibrosis. A further methodological question is the potentially limited external validity of the results for different populations and settings. Our study included a cohort of Italian NAFLD patients, largely obese, at high prevalence of NASH and severe fibrosis, who may be different, in terms of both metabolic features and severity of liver disease, from the majority of prevalent cases of NAFLD in the general population. The lack of data on a control Sicilian population might further limit the strength of our results; however higher prevalence of vascular damage in subjects with NAFLD seem to be a finding potentially applicable to other italian cohorts.

Another limitation is that our study included a cohort of Italian NAFLD patients referred to a tertiary referral center, largely obese, with abnormal ALT levels, and at high prevalence of NASH and severe fibrosis, who may be different, in terms of metabolic features, ALT levels and severity of liver disease, from the majority of prevalent cases of NAFLD in the general population”.

Finally, we also need data on serum levels and liver expression of proinflammatory and profibrogenic cytokines potentially involved in vascular changes of NAFLD patients.

## Conclusions

This study reports that NAFLD subjects in comparison to controls without NAFLD had: (1) lower mean values of RHI; (2) higher mean values of PWV and Aix; (3) lower MMSE; (4) a significant relationship at multivariate analysis between RHI and MMSE with NAFLD, (5) a significant negative relationship between ballooning grade and MMSE grade only in NASH subjects; (6) a significant positive relationship between steatosis and augmentation index only in NASH subjects.

Several previous studies [54–56] pinpointed the inflammatory background of nonalcoholic fatty liver disease, thus future studies will be addressed to evaluate the relationship between inflammatory markers and arterial stiffness and endothelial function indexes in NAFLD subjects.

Future research could address these issues both by means of longitudinal studies to evaluate incidence of new-onset cardiovascular events and their connection with arterial stiffness, endothelial and cognitive markers. They also should analyze the beneficial effects of cardiovascular drugs such as statins and ACE inhibitors on these surrogate markers (indicators of arterial stiffness, endothelial function and cognitive performance) in NAFLD subjects.
